# Additive clinical impact of epidermal growth factor receptor and podocalyxin-like protein expression in pancreatic and periampullary adenocarcinomas

**DOI:** 10.1038/s41598-020-67187-z

**Published:** 2020-06-25

**Authors:** Margareta Heby, Emelie Karnevi, Jacob Elebro, Björn Nodin, Jakob Eberhard, Kapo Saukkonen, Jaana Hagström, Harri Mustonen, Hanna Seppänen, Caj Haglund, Karin Jirström, Anna H. Larsson

**Affiliations:** 1 Department of Clinical Sciences Lund, Division of Oncology and Pathology, Lund University, Skåne University Hospital, 22185 Lund, Sweden; 2 Department of Surgery, University of Helsinki and Helsinki University Hospital, P.O. Box 440, FIN-00029 HUS, Helsinki, Finland; 3 Research Programmes Unit, Translational Cancer Biology, University of Helsinki, P.O. Box 63, FIN-00014 University of Helsinki, Helsinki, Finland; 4 Department of Pathology, Haartman Institute and HUSLAB, University of Helsinki and Helsinki University Hospital, Helsinki, FIN-00014 University of Helsinki, Helsinki, Finland

**Keywords:** Biomarkers, Oncology

## Abstract

The outcome of periampullary adenocarcinomas remains poor with few treatment options. Podocalyxin-like protein (PODXL) is an anti-adhesive protein, the high expression of which has been shown to confer a poor prognosis in numerous malignancies. A correlation and adverse prognostic synergy between PODXL and the epidermal growth factor receptor (EGFR) has been observed in colorectal cancer. Here, we investigated whether this also applies to periampullary adenocarcinomas. We analyzed the immunohistochemical expression of PODXL and EGFR in tissue microarrays with tumors from two patient cohorts; (Cohort 1, n = 175) and (Cohort 2, n = 189). The effect of TGF-β-induced expression and siRNA-mediated knockdown of PODXL and EGFR, were investigated in pancreatic cancer cells (PANC-1) *in vitro*. We found a correlation between PODXL and EGFR in these cancers, and a synergistic adverse effect on survival. Furthermore, silencing PODXL in pancreatic cancer cells resulted in the down-regulation of EGFR, but not vice versa. Consequently, these findings suggest a functional link between PODXL and EGFR, and the potential combined utility as biomarkers possibly improving patient stratification. Further studies examining the mechanistic basis underlying these observations may open new avenues of targeted treatment options for subsets of patients affected by these particularly aggressive cancers.

## Introduction

The periampullary region includes the head of the pancreas, the distal bile duct, the ampulla of Vater and the periampullary duodenum. Adenocarcinomas developing in this region represent a heterogeneous group of tumors, wherein morphology appear to provide more prognostic information than the tumor origin, in that pancreatobiliary type (PB-type) differentiation is associated with a significantly shorter survival than intestinal type (I-type)^1,2^. Pancreatic cancer, the most common type of periampullary adenocarcinomas, has a 5-year overall survival of merely 7%^3^, the dismal prognosis partly due to a high rate of advanced tumor stages at the time of diagnosis^4^.

Podocalyxin-like protein (PODXL) is a member of the CD34 family of transmembrane glycoproteins consisting of a mucin-like extracellular domain and a cytoplasmic tail. PODXL is expressed on podocytes and the apical surface of glomerular epithelial cells of the kidney^5^, where it plays an important role in maintaining adequate filtration^6^, due to its anti-adhesive properties. Moreover, PODXL is involved in the regulation of cell adhesion and is expressed for instance on vascular endothelia^7^ and hematopoietic progenitor cells^8,9^. Aside from these normal conditions, PODXL has been found to be upregulated in various malignancies, and its strong expression, particularly in the cell membrane, associates with a more aggressive tumor phenotype and a poor prognosis in periampullary^10^, breast^11^, colorectal^12–15^ ovarian^16^, and bladder cancers^17^, as well as in glioblastoma^18^. The role of PODXL in tumorigenesis is unclear, but evidence suggests that PODXL is involved in epithelial-mesenchymal transition (EMT), a process by which epithelial cells acquire invasive and migratory properties and make them resistant to apoptosis^19^. EMT is regulated by various differentiation and growth factors, most importantly transforming growth factor β (TGF-β). When TGF-β is added to epithelial cells in culture, EMT will be induced^20^.

The epidermal growth factor receptor (EGFR) is a glycoprotein and a tyrosine kinase receptor, which belongs to the human epidermal growth factor receptor (HER) family. EGFR activation induces intracellular signaling-cascades important for growth, differentiation and cell survival^21^, all essential for tumor development. EGFR overexpression is common in several malignancies including cancers of the breast, ovary, bladder, kidney, lung and brain^21^. In pancreatic cancer, EGFR has been found to be overexpressed in 30–95% of the cases^22,23^ and associated with a poor prognosis^24^. Previous work has shown that high expression of PODXL and EGFR impair survival in colorectal cancer in a synergistic way^25^. This study thus aimed to investigate the relationship between the expression of PODXL and EGFR in periampullary adenocarcinomas, including pancreatic cancer, with focus on morphological subtypes. We also examined the functional interplay between PODXL and EGFR in pancreatic cancer cells *in vitro*.

## Methods

### Patients

#### Cohort 1

Cohort 1 is a previously described retrospective consecutive cohort of 175 patients with primary periampullary adenocarcinomas who were surgically treated with pancreaticoduodenectomy at the University hospitals of Lund and Malmö, Sweden, from 1 January 2001 through 31 December 2011^10,26–29^. Morphologically, 110 cases were classified as PB-type and 65 cases as I-type adenocarcinomas. Fortysix of these 175 patients presented with pancreatic ductal adenocarcinoma. Data on survival were gathered from the Swedish National Civil Register and clinical data was obtained retrospectively from medical records. Follow-up began on the date of surgery and ended upon death or upon the final update in March 2017. Patients were identified through broad searches in the pathology database, and all haematoxylin and eosin (H&E) stained slides were re-evaluated by one pathologist (JEL), blinded to the original report and outcome, with the decision on tumor origin and morphological type being based on several criteria, as previously described^26^. The Ethics Committee of Lund University (ref no. 445/07) approved the study, whereby the committee waived the need for consent other than by opting out. Methods were carried out in accordance with relevant guidelines and regulations.

#### Cohort 2

Cohort 2 is a previously described retrospective cohort of 189 consecutive patients with pancreatic ductal adenocarcinoma undergoing surgery at the Department of Surgery, Helsinki University Hospital in Finland from 1 January 2000 through 31 December 2011^30,31^. A majority of the tumors were of periampullary origin, however a small number of the tumors originated in cauda pancreatis. Patients who received neoadjuvant chemotherapy (n = 21) were excluded from the study. Data on survival and cause of death were gathered from medical records, Statistics Finland, and the Finnish Population Registry. The Surgical Ethics Committee of Helsinki University Central Hospital (Dnro HUS 226/E6/06, extension TMK02 §66 17.4.2013) approved the study. Permission to use tissue samples without individual informed consent was granted by the National Supervisory Authority of Health and Welfare (Valvira Dnro 10041/06.01.03.01/2012). Methods were carried out in accordance with relevant guidelines and regulations.

### Tissue microarray construction

As previously described for Cohort 1^10^, a semi-automated arraying device (TMArrayer, Pathology Devices, Westminister, MD, USA) was used for the construction of tissue microarrays (TMAs). Three tissue cores of 1 mm were sampled from each of the 175 primary tumors and from lymph node metastases from 105 of the cases. In addition, two tissue cores (1 mm) were obtained from adjacent benign-appearing pancreatic tissue in 50 cases with duodenal or ampullary cancer.

For Cohort 2, TMAs were constructed using a semiautomatic tissue microarrayer (Tissue Arrayer 1, Beecher Instruments Inc., Silver Spring, MD, USA). Two 1-mm cores were taken from representative areas of each of the 168 tumors. Fifteen cases were excluded from analysis due to inadequate or missing cores. All samples were re-evaluated by two experienced pathologists. Histological images of the tumor tissues from both cohorts were published elsewhere^10,28,30^.

### Immunohistochemistry and staining evaluation

For immunohistochemical analysis of PODXL expression in Cohort 1, TMA sections of 4 μm were automatically pre-treated using the PT Link system, and then stained in an Autostainer Plus (DAKO; Glostrup, Copenhagen, Denmark) with a polyclonal anti-PODXL antibody (HPA2110, Atlas Antibodies AB, Stockholm, Sweden, diluted to 1: 250), as described elsewhere^10^. The expression of PODXL was recorded as negative (0), weak cytoplasmic positivity in any proportion of cells (1), moderate cytoplasmic positivity in any proportion of cells (2), distinct membranous positivity in ≤50% of cells (3) and distinct membranous positivity in>50% of cells (4)^10,12–14,17^, and dichotomized into low (0–2) or high (3–4). Staining evaluation was performed by two independent observers (MH and KJ), who were blinded to the clinical and outcome data.

Immunohistochemical technique and PODXL staining evaluation in Cohort 2 were described elsewhere^30^. The same polyclonal antibody (HPA 2110, Atlas Antibodies, Stockholm, Sweden) was used as that in Cohort 1.

For immunohistochemical analysis of EGFR in both cohorts, 4 μm TMA-sections were pre-treated using the PT-link system (Dako), and subsequently stained with a monoclonal anti-EGFR antibody (31G7, Zymed Laboratories Inc, San Francisco, CA, USA, diluted to 1:25) using the Autostainer Plus (Dako, Glostrup, Denmark). The evaluation of the EGFR protein expression in Cohort 1 was described elsewhere^28^ but was now performed on Cohort 2. The evaluation was performed using the recommended protocol for HER2 testing in gastric and gastroesophageal junction cancer biopsies^32^, taking complete, basolateral, or lateral membranous reactivity in a minimum of five clustered positive cancer cells into account. The intensity recorded was rated as 0, 1+, 2+ or 3+. Protein expression was grouped into low (0–2+) or high (3+) expression, as described elsewhere^28^. Cytoplasmic staining was grouped as 1+ in the statistical analyses. Staining evaluation was performed by three independent observers (MH, AL and KJ). Any inter-observer discrepancies were discussed in order to reach consensus. In Cohort 2, 10 cases were excluded from the analyses due to staining or interpretation problems with the EGFR antibody.

### Cell culture

Human pancreatic cancer cell lines PANC-1 and human foetal foreskin fibroblasts (HFFF2) were purchased from Sigma-Aldrich and the growth medium, foetal bovine serum (FBS) and antibiotics were obtained from Nordic Biolabs. The cells were maintained in an RPMI1640 or DMEM medium supplemented with 10% FBS and antibiotics (100 U/ml penicillin and 100 μg/ml streptomycin) in a humidified 5% CO_2_ atmosphere at 37°C. All *in vitro* reagents were purchased from ThermoFisher Scientific unless stated otherwise. All *in vitro* experiments were repeated at least three times.

### siRNA transfection

Pancreatic cancer cells were seeded in T-25 flasks (5×10^5^ cells) and incubated for 72 hours in 37°C for siRNA transfection. Subsequently, the cells were washed twice with phosphate buffered saline (PBS) and received the medium without FBS, together with lipofectamine 2000 and the negative control, anti-PODXL (s10770 + s10771) or anti-EGFR (s563 + s565) siRNA in OptiMEM to a final siRNA concentration of 25 nM. After 4.5 hours, the transfection was stopped, and the medium changed to a full-growth medium. The cells were then left to recover overnight, and the next day, cells were harvested and spun down to pellets. The pellets were either resuspended in Trizol and stored at −20°C for qPCR, or fixated, dehydrated and embedded in paraffin for immunohistochemistry.

### TGF- β incubation

For TGF-β incubation, pancreatic cancer cells were seeded in T-25 flasks (5×10^5^ cells), incubated for 72 hours at 37°C, and then incubated with TGF-β (10 ng/ml) for 48 hours. Following this, the cells were harvested and spun down to pellets. The pellets were either resuspended in Trizol and stored at −20°C for qPCR, or fixated, dehydrated and embedded in paraffin for immunohistochemistry.

### Organotypic assay

The method of designing an organotypic assay was described elsewhere^33^. Twenty-four hours after siRNA transfection or TGF-β incubation, the 3D organotypic model was prepared according to Moutasim *et al*. and Froeling *et al*.^34,35^. Prior to making the gel (7 parts Collagen I, 1 part DMEM 10×, 1 part FBS and 1 part regular DMEM growth medium), inserts (3 μm pores) in a 24-wells plate were coated with collagen. The gel was made from 3.5 parts Collagen I, 3.5 parts Matrigel, 1 part DMEM 10×, 1 part FBS and 1 part cell suspension (containing 2.5×10^4^ HFFF2 cells) and added to each collagen-coated insert. The gels were left to incubate for 1 hour at 37 C, and following gel polymerization, the cancer cells (5×10^4^) were harvested and mixed with HFFF2 (2.5×10^4^) and seeded on top of the gels. Subsequently, the medium was added to the wells and the organotypic model was incubated overnight at 37 C. After overnight incubation, the medium was removed from the wells and inserts and the medium was added to the well up to the bottom of the insert to create an air-liquid interface. Every two to three days, the medium was changed, and after incubation for seven days, the gels were fixated in 4% paraformaldehyde and paraffin embedded. Sections (3-μm-thick) of the gels were stained with H&E for the visualization of cancer cell invasion. For TGF-β incubated cells prior to the model, gel sections were also stained for PODXL or EGFR using the same antibodies as those used for tissue samples. Representative pictures of one out of three individual repeats were taken with cellSens dimension software at 20x magnification. The rate of invasion in the gel was based on estimates of the observed number of cells entering the gel, and not formally quantified.

### Immunohistochemistry

TMAs were constructed from the paraffin-embedded cell pellets, and the staining of PODXL and EGFR was performed in the same manner as the tissue samples. The cell pellets were used to visualize protein expression under the different conditions tested *in vitro*.

### qPCR

The method of qPCR has been described elsewhere^33^. To remove the cell debris, the cell samples were thawed and spun down. According to the manufacturer’s instructions, RNA purification was performed using Trizol with phasemaker tubes. Thereafter, RNA cleanup was performed using the RNeasy minelute kit (QIAGEN) and the RNA concentration was determined using Qubit with the RNA HS kit. cDNA reverse transcription was performed using the high-capacity cDNA reverse transcription kit, prior to qPCR, and the total cDNA concentration was determined using Qubit with the DNA HS kit. Ten ng per reaction of each sample was used to run qPCR with the PODXL or EGFR TaqMan gene expression assay (Assay ID Hs01574644_m1 and Hs01076090_m1, respectively), with samples run in triplicates. 18 S served as the endogenous control (Assay ID Hs039288985_g1).

### Statistical analysis

In Cohort 1, three patients were excluded from the survival analyses; two with I-type adenocarcinomas who died within one month of surgery due to complications and one with PB-type adenocarcinoma who emigrated five months after surgery. Another two patients with PB-type adenocarcinomas who had received neoadjuvant chemotherapy were excluded from the survival analyses since the treatment might have affected the expression of PODXL. The Kaplan Meier analysis and log-rank test were applied to illustrate differences in overall survival (OS) with respect to PODXL and EGFR expression. The p-value was based on a pairwise comparison. Cox regression proportional hazard models were used to estimate hazard ratios (HRs) for death in both univariable and multivariable analysis, adjusted for tumor grade, T-stage (T1–2 vs. T3–4), N-stage (N0 vs. N1), perineural growth, blood vessel invasion, lymphatic invasion, the invasion of peripancreatic fat, adjuvant treatment, and for morphological type in Cohort 1, and adjusted for tumor grade, T-stage (T1–2 vs. T3–4), N-stage (N0 vs. N1), perineural growth, blood vessel invasion, and adjuvant treatment in Cohort 2. The proportional hazard (PH) assumption was tested graphically by using log-minus-log plots. The PH assumption was also evaluated using the Cox regression analysis with a time-dependent covariate analysis, whereby the PH assumption was considered to be satisfied when the factor-by-time interaction was not significant. All statistical analyses were performed using IBM SPSS Statistics version 22.0 (SPSS Inc., Chicago, IL, USA). All tests were two-sided and we considered p-values <0.05 significant.

## Results

### PODXL and EGFR expression

**Figure 1:**
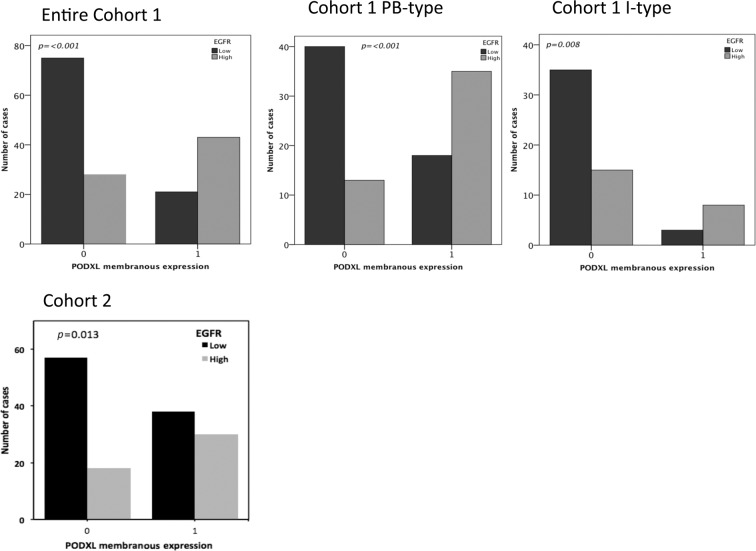
Correlations between the immunohistochemical expression of PODXL and EGFR. Bar charts visualizing the correlations between low vs. high (0 vs. 1) PODXL and low vs. high (0 vs. 1) EGFR. The top row shows the correlations for Cohort 1, for the entire cohort and divided into morphological subtypes. The bottom row shows the correlations for Cohort 2.

A significant correlation between the expression of PODXL and EGFR was identified in both cohorts, as well as in the stratified analysis according to morphological subtype (Fig. 1). The associations of clinicopathological factors with PODXL and EGFR expressions in Cohort 1^10,28^ and with PODXL in Cohort 2^30^ were described elsewhere.

### Clinical outcome related to the combined strata of PODXL and EGFR expression

#### Cohort 1

**Figure 2:**
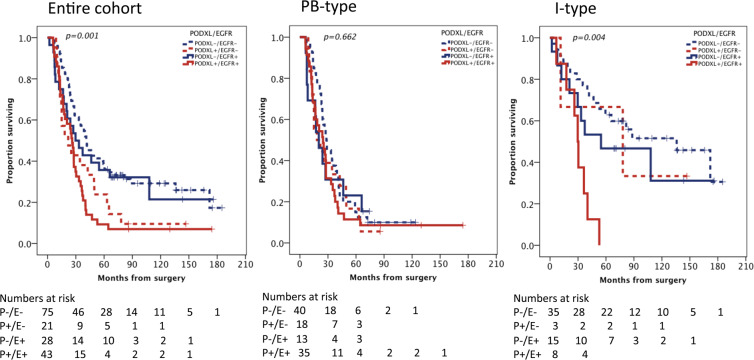
Overall survival according to PODXL and EGFR expressions in Cohort 1. Kaplan-Meier estimates for the overall survival according to the combined strata of low and high PODXL and EGFR expression in Cohort 1, shown for the entire cohort, for PB-type and I-type tumors. The p-value was based on a pairwise comparison with PODXL-/EGFR- and PODXL+/EGFR+.

As Fig. 2 shows, the Kaplan-Meier analysis revealed that patients with tumors displaying high levels of both PODXL and EGFR exhibited the shortest OS in the entire cohort and in tumors with an I-type morphology. This was, however, not seen in PB-type tumors. As shown in Table 1, the high PODXL expression served as an independent predictor of a diminished OS across the entire cohort, among both patients with low EGFR tumors (HR 1.84; 95% CI 1.09–3.12), and among patients with high EGFR tumors (HR 1.78; 95% CI 1.03–3.07). The highest risk of death was observed among patients with I-type tumors displaying a high expression of both PODXL and EGFR (unadjusted HR 2.90; 95% CI 1.03–8.18), and this association remained significant in the adjusted analysis (HR 4.77; 95% CI 1.02–22.33). We considered the PH assumption satisfied using the graphic evaluation of log-minus-log plots (data not shown). Moreover, the time-dependent covariate was not significant for PODXL and EFGR, and therefore, the factor x time interaction term was dropped from the model.

**Table 1:** Unadjusted and adjusted hazard ratios of overall survival according to PODXL and EGFR expression in Cohort 1.

	All			Pancreatobiliary type			Intestinal type		
		Unadjusted	Adjusted		Unadjusted	Adjusted		Unadjusted	Adjusted
	n (events)	HR (95%CI)	HR (95%CI)	n (events)	HR (95%CI)	HR (95%CI)	n (events)	HR (95%CI)	HR (95%CI)
EGFR low									
PODXL low	75 (54)	1.00	1.00	40 (36)	1.00	1.00	35 (18)	1.00	1.00
PODXL high	21 (19)	**1.84 (1.09–3.12)**	1.23 (0.69–2.17)	18 (17)	1.24 (0.69–2.22)	1.15 (0.62–2.14)	3 (2)	1.43 (0.33–6.21)	1.00 (0.11–9.08)
EGFR high									
PODXL low	28 (20)	1.00	1.00	13 (11)	1.00	1.00	15 (9)	1.00	1.00
PODXL high	43 (40)	**1.78 (1.03–3.07)**	1.25 (0.61–2.60)	35 (32)	1.12 (0.56–2.24)	0.79 (0.31–1.97)	8 (8)	**2.90 (1.03–8.18)**	**4.77 (1.02–22.33)**

Multivariable analysis adjusted for tumor morphology (entire cohort), tumor grade, T-stage (T1–2 vs. T3–4), N-stage (N0 vs. N1), perineural growth, lymphatic invasion, blood vessel invasion, invasion of peripancreatic fat and adjuvant treatment. Statistically significant values displayed in bold text.

#### Cohort 2

**Figure 3:**
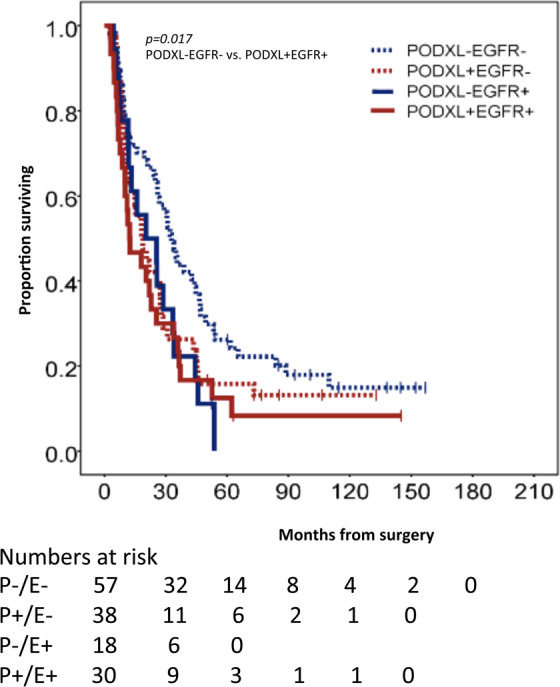
Overall survival according to PODXL and EGFR expressions in Cohort 2. Kaplan-Meier estimates for the overall survival in pancreatic ductal adenocarcinoma according to the combined strata of low and high expression of PODXL and EGFR in Cohort 2. The p-value was based on a pairwise comparison with PODXL-/EGFR- and PODXL+/EGFR+.

As demonstrated in Fig. 3, the Kaplan-Meier analysis found that a high PODXL expression, in combination with a high EGFR expression, correlated with a significantly reduced overall survival (p = 0.017) compared to patients without the overexpression of the two proteins. These results mirror those in Cohort 1. However, as shown in Table 2, a high PODXL expression did not associate with a reduced overall survival, irrespective of EGFR expression.

**Table 2:** Unadjusted and adjusted hazard ratios of overall survival according to PODXL and EGFR expression in Cohort 2.

	n (events)	Unadjusted	Adjusted
		HR (95%CI)	HR (95%CI)
**EGFR low**			
PODXL low	57 (44)	1.00	1.00
PODXL high	38 (31)	1.48 (0.94–2.31)	1.27 (0.75–2.16)
**EGFR high**			
PODXL low	18 (16)	1.00	1.00
PODXL high	30 (26)	1.05 (0.57–1.93)	1.25 (0.59–2.65)

Multivariable analysis adjusted for tumor grade, T-stage (T1–2 vs. T3–4), N-stage (N0 vs. N1), perineural growth, blood vessel invasion and adjuvant treatment. Statistically significant values displayed in bold text.

### Effects of TGF-β incubation and the siRNA-mediated silencing of PODXL and EGFR in pancreatic cancer cells *in vitro*

**Figure 4:**
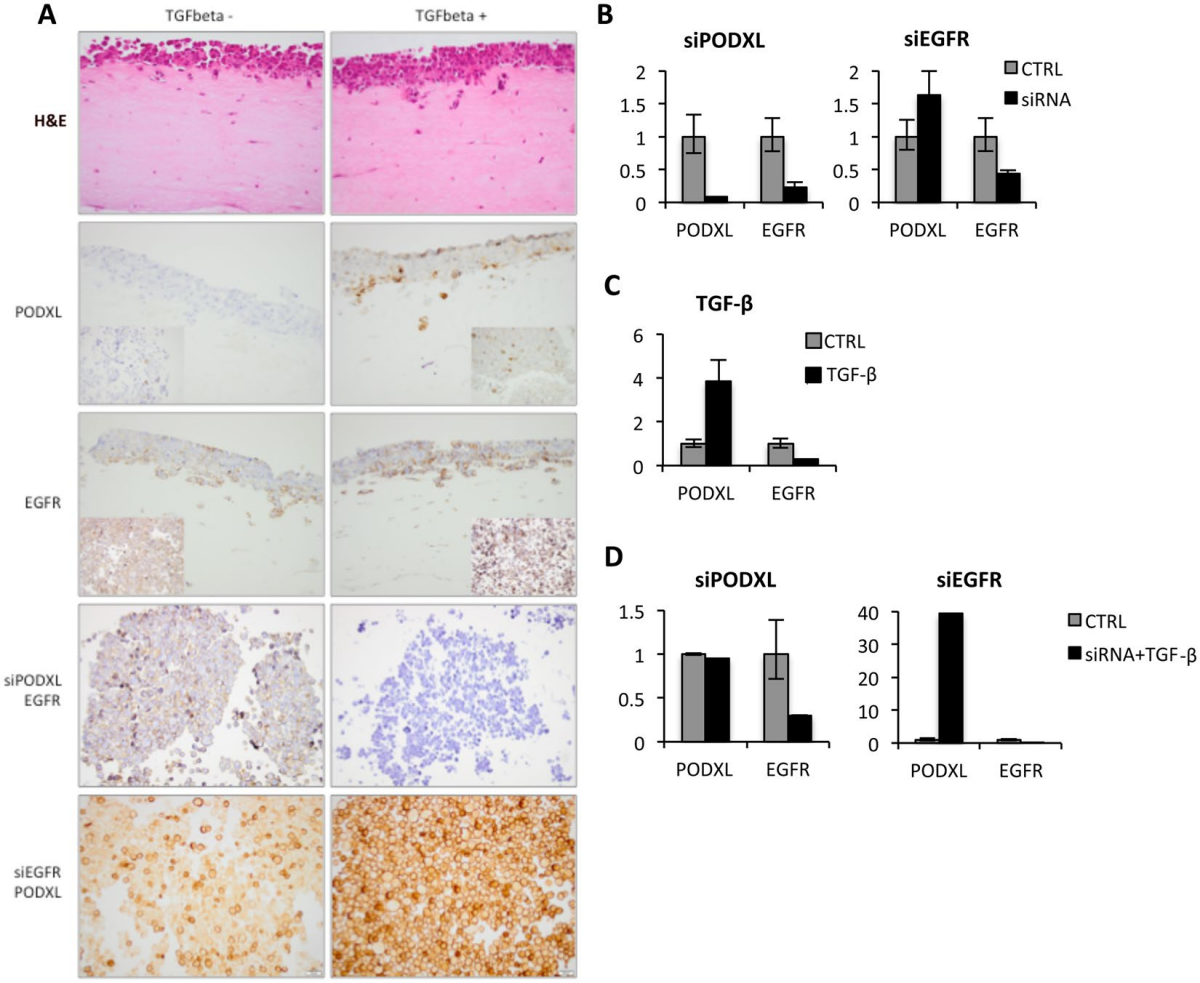
Effects of TGF-β incubation and siRNA-mediated silencing of *PODXL* and *EGFR* in pancreatic cancer cells. (**A**) 3D organotypic model of the PANC-1 cell line on gel sections, as visualized in an H&E stain (top row), without (left column) and after (right column) incubation with TGF-β. As shown in the second row, the expression of PODXL increases upon incubation with TGF-β, particularly along the invasive front (inserts show the immunocytochemistry on cell pellets). The third row shows that the expression of EGFR does not markedly change upon incubation with TGF-β. The fourth row shows the EGFR protein expression in siPODXL silenced PANC-1 cells and the bottom panel shows PODXL protein expression in siEGFR silenced PANC-1 cells. (**B**) qPCR demonstrating the mRNA levels of PODXL and EGFR in siPODXL and siEGFR PANC-1 cell line. (**C**) qPCR demonstrating the mRNA levels of PODXL and EGFR in TGF-β incubated PANC-1 cell line. (**D**) qPCR demonstrating mRNA levels of PODXL and EGFR where PANC-1 cells were incubated with TGF-β following siRNA transfection.

In addition to the results found in both cohorts, functional studies were performed *in vitro* using PANC-1 pancreatic cancer cells either transfected with siRNA against PODXL (siPODXL) or EGFR (siEGFR), or incubated with TGF-β. Figure 4A displays the protein expression under different conditions in the 3D organotypic model and cell pellets. In the top row, where cells were incubated with TGF-β alone, we found an increasing invasion following TGF-β incubation. This was visualized in the H&E staining by the greater number of cells entering the gel compared to the control. In addition, the expression of PODXL was more prominent along the invasive front, however, the protein expression of EGFR was fairly unchanged (second and third row; inserts show immunocytochemistry on the cell pellets). Furthermore, the mRNA levels of PODXL and EGFR were successfully transiently knocked down in PANC-1 cells by siRNA (Fig. 4B). Here, the siPODXL-transfected cells were reduced in both PODXL and EGFR, whereas the siEGFR-transfected cells alone appeared reduced in EGFR. Following TGF-β incubation, the PODXL mRNA levels showed a fourfold increase and EGFR was somewhat reduced (Fig. 4C). To examine the combined effect of TGF-β and siRNA on PODXL and EGFR expression, PANC-1 cells were incubated with TGF-β following siRNA transfection (bottom two rows of Fig. 4A, and Fig. 4D). As shown in Fig. 4A, EGFR expression disappeared following the combination of siPODXL and TGF-β, whereas PODXL expression increased after the combination of siEGFR and TGF-β. This was also visible at the mRNA level, where the combination of siPODXL and TGF-β incubation resulted in a reduction in EGFR, and siEGFR together with TGF-β greatly enhanced the PODXL expression (Fig. 4D). This confirms that PODXL appears to influence EGFR expression but not vice versa and, furthermore, that PODXL suppression can prevent the overexpression induced by TGF-β.

## Discussion

The prognosis for patients with pancreatic and other periampullary adenocarcinomas remains poor with few treatment options. Thus, identifying biomarkers to better understand and define this group of tumors in a clinically relevant context is important. In this translational study we investigated PODXL and EGFR and demonstrated a significant relationship between the overexpression of these proteins in pancreatic and other periampullary adenocarcinomas. In the cohort comprising the full range of periampullary cancers, this association was particularly evident in I-type tumors, whereas in PB-type tumors, all patients exhibited a similarly poor prognosis. However, in Cohort 2, which comprised only ductal pancreatic cancer, patients with tumors overexpressing both PODXL and EGFR exhibited a significantly shorter overall survival. The association between the overexpression of PODXL and EGFR seen in I-type periampullary tumors was not confirmed in PB-type tumors or in pancreatic cancer in Cohort 2 in the Cox regression analysis, further indicating that I-type tumors resemble and should be treated as colorectal cancer^25^.

The results from the tumor analyses were further verified through the *in vitro* data. Incubation with TGF-β rendered pancreatic cancer cells seemingly more invasive, leading to an increased expression of PODXL, particularly at the invasive front. This confirms that PODXL is markedly increased during TGF-β-induced EMT, a process associated with cell migration and increased invasion, as previously described by e.g. Meng *et al*.^19^ and Fröse *et al*.^36^. In addition, TGF-β has been associated with EMT in pancreatic cancer^37^, representing a central pathway in the development of pancreatic cancer^38–40^. The influence of TGF-β on PODXL and tumor aggressiveness might provide an explanation of the mechanism indicative of the association between high PODXL and reduced survival among patients. For instance, a study by Javle *et al*.^41^ demonstrated that a high plasma level of TGF-β1 was associated with diminished survival among patients with advanced pancreatic cancer. Notably in our study, while the knockdown of PODXL reduced EGFR expression, PODXL levels were not affected by the knockdown of EGFR. This was further confirmed in the presence of TGF-β, where siPODXL prevented an increase in PODXL and EGFR but siEGFR played no effect on protein expression. This agrees with our findings in both cohorts, where high PODXL expressing tumors were also more likely to display high levels of EGFR. Furthermore, EGFR signaling has been shown to take part in cancer progression, in that EGFR overexpression leads to enhanced invasiveness through the loss of E-cadherin^42^. Accordingly, inhibiting EGFR may affect EMT and inhibit cancer cell migration, although the association between EGFR and EMT is not as apparent as it is for PODXL. Moreover, the relationship between PODXL and EGFR is not fully understood, but previous *in vitro* studies have demonstrated a possible interaction through the Na^+^/H^+^ Exchanger Regulatory Factor (NHERF) proteins. Expression of PODXL leads to the induction of the NHERF proteins to the surface of the epithelial cell^43^. On the cell surface, NHERF-1 has been demonstrated to stabilize EGFR, thus restricting receptor downregulation, leading to enhanced EGFR signaling^44^.

The response to EGFR inhibitors in e.g. non-small cell lung cancer depends upon specific EGFR mutations^45^, which are rare in pancreatic cancer^22^. Furthermore, in a phase III trial among patients with locally advanced or metastatic pancreatic cancer, the EGFR expression did not predict the response to the EGFR tyrosine kinase inhibitor erlotinib, when added to gemcitabine^46^. In addition, a study by Izumchenko *et al*.^47^ demonstrated a mechanism of how resistance to erlotinib may develop. During TGF-β-mediated EMT the microRNAs 200 (miR200) family was inhibited and the expression of the mitogen-inducible gene 6 (MIG6), which is a negative regulator of EGFR, was upregulated, thus leading to resistance to erlotinib. The interaction between PODXL and EGFR and the potential role of PODXL in upregulating EGFR found in the present study is noteworthy, and it would be of interest to explore if PODXL expression affects the response to anti-EGFR directed therapy.

Since periampullary cancers encompass several different tumor origins, a pancreatic cancer cell line was chosen for the *in vitro* portion of this study. This decision was made based on the fact that pancreatic cancer represented the largest subset of cancers among the periampullary tumors in Cohort 1, whereby Cohort 2 only includes ductal pancreatic cancers. The cell line, PANC-1, used here was obtained from a ductal pancreatic carcinoma from a 56-year old Caucasian male^48^. The tumor cells are *TP53*- and *KRAS*- mutated, but *SMAD-4* wild type. Mutations in the tumor suppressor gene *TP53* and the proto-oncogene *KRAS* lead to cell cycle deregulation, inhibition of apoptosis, cell invasion, metastasis and poor treatment response, all essential in tumor development. Other cell lines currently used to study pancreatic carcinogenesis include MIA PaCa-2 and BxPC-3. When designing this study, these other cell lines were considered. However, the MIA PaCa-2 cell line has an impaired TGF-βR2 status, thus rendering it unsuitable for the experimental setting with TGF-β stimulation used herein, and BxPC-3 is *KRAS* wild-type^48^. Since *KRAS* mutations occur in almost all primary tumors of pancreatic cancer, and appear early in the progression of the disease, this cell line was also deemed unsuitable^49^.

One strength of this study stems from its use of two well-characterized, consecutive, and comparatively large patient cohorts with periampullary adenocarcinomas, including pancreatic cancer, both spanning over the same period of time. The cohorts were kept separate whereby the use of two different cohorts can be considered a validation of the results. Furthermore, given the fact that differentiation of tumor origin can be difficult in the clinical setting, using the morphological type in treatment decisions is crucial. All histological samples were re-evaluated by experienced pathologists, and clinical follow-up and survival data were reliable and up-to-date.

A potential limitation to this study lies in the use of TMA, since this technique, although being a well-validated method^50^, may not reflect heterogeneous biomarker expression. However, the analysis of one whole tissue section will also merely reflect a small portion of the tumor, and sampling of tissue cores from multiple donor blocks, carried out in this study, may provide a more representative picture of the entire tumor.

We have previously demonstrated that patients with I-type, but not PB-type, tumors displaying high expression of PODXL benefitted from adjuvant chemotherapy^10^. Furthermore, in patients with PB-type tumors receiving adjuvant gemcitabine, high expression of EGFR was significantly associated with a shorter overall survival and recurrence free survival, with a significant treatment interaction in relation to OS^28^. These findings suggest that adjuvant treatment has a limited or no effect on survival in patients with PB-type tumors with a high EGFR and PODXL expression. Speculatively, the inhibition of EGFR might serve as an alternative treatment option for these patients.
